# Author Correction: Genetic regulation of serum IgA levels and susceptibility to common immune, infectious, kidney, and cardio-metabolic traits

**DOI:** 10.1038/s41467-023-36340-3

**Published:** 2023-02-06

**Authors:** Lili Liu, Atlas Khan, Elena Sanchez-Rodriguez, Francesca Zanoni, Yifu Li, Nicholas Steers, Olivia Balderes, Junying Zhang, Priya Krithivasan, Robert A. LeDesma, Clara Fischman, Scott J. Hebbring, John B. Harley, Halima Moncrieffe, Leah C. Kottyan, Bahram Namjou-Khales, Theresa L. Walunas, Rachel Knevel, Soumya Raychaudhuri, Elizabeth W. Karlson, Joshua C. Denny, Ian B. Stanaway, David Crosslin, Thomas Rauen, Jürgen Floege, Frank Eitner, Zina Moldoveanu, Colin Reily, Barbora Knoppova, Stacy Hall, Justin T. Sheff, Bruce A. Julian, Robert J. Wyatt, Hitoshi Suzuki, Jingyuan Xie, Nan Chen, Xujie Zhou, Hong Zhang, Lennart Hammarström, Alexander Viktorin, Patrik K. E. Magnusson, Ning Shang, George Hripcsak, Chunhua Weng, Tatjana Rundek, Mitchell S. V. Elkind, Elizabeth C. Oelsner, R. Graham Barr, Iuliana Ionita-Laza, Jan Novak, Ali G. Gharavi, Krzysztof Kiryluk

**Affiliations:** 1 Division of Nephrology, Department of Medicine, Vagelos College of Physicians & Surgeons, Columbia University, New York, NY USA; 2 Lewis Thomas Laboratory, Department of Molecular Biology, Princeton University, Princeton, NJ USA; 3 Department of Medicine, University of Pennsylvania, Philadelphia, PA USA; 4 Center for Human Genetics, Marshfield Clinic Research Institute, Marshfield, WI USA; 5 Center of Autoimmune Genomics and Etiology, Cincinnati Children’s Hospital, Cincinnati, OH USA; 6 Department of Pediatrics, University of Cincinnati College of Medicine, Cincinnati, OH USA; 7 US Department of Veterans Affairs Medical Center, Cincinnati, OH USA; 8 Department of Medicine, Northwestern University Feinberg School of Medicine, Chicago, IL USA; 9 Division of Rheumatology, Immunology and Allergy, Brigham and Women’s Hospital and Harvard Medical School, Boston, MA USA; 10 Department of Medicine, Vanderbilt University School of Medicine, Nashville, TN USA; 11 Kidney Research Institute, Division of Nephrology, Department of Medicine, University of Washington, Seattle, WA USA; 12 Department of Biomedical Informatics and Medical Education, School of Medicine, University of Washington, Seattle, WA USA; 13 Department of Nephrology, RWTH University of Aachen, Aachen, Germany; 14 Kidney Diseases Research, Bayer Pharma AG, Wuppertal, Germany; 15 Department of Microbiology and Medicine, University of Alabama at Birmingham, Birmingham, AL USA; 16 Division of Pediatric Nephrology, University of Tennessee Health Sciences Center, Memphis, TN USA; 17 Department of Nephrology, Juntendo University Faculty of Medicine, Tokyo, Japan; 18 Department of Nephrology, Institute of Nephrology, Shanghai Ruijin Hospital, Shanghai Jiao Tong University School of Medicine, Shanghai, China; 19 Renal Division, Peking University First Hospital, Peking University Institute of Nephrology, Beijing, China; 20 Department of Biosciences and Nutrition, Karolinska Institutet, Stockholm, Sweden; 21 Department of Medical Epidemiology and Biostatistics, Karolinska Institutet, Stockholm, Sweden; 22 Department of Biomedical Informatics, Vagelos College of Physicians & Surgeons, Columbia University, New York, NY USA; 23 Department of Neurology, University of Miami, Miami, FL USA; 24 Evelyn F. McKnight Brain Institute, University of Miami, Miami, FL USA; 25 Department of Neurology, Vagelos College of Physicians & Surgeons, Columbia University, New York, NY USA; 26 Division of General Medicine, Department of Medicine, Vagelos College of Physicians & Surgeons, Columbia University, New York, NY USA; 27 Department of Epidemiology, Mailman School of Public Health, Columbia University, New York, NY USA; 28 Department of Biostatistics, Mailman School of Public Health, Columbia University, New York, NY USA

**Keywords:** Genome-wide association studies, Mucosal immunology, IgA nephropathy

Correction to: *Nature Communications* 10.1038/s41467-022-34456-6, published online 11 November 2022

The original version of this Article contained two errors in table 1. The correct version of the 13^th^ row of the first column states “Chinese (East Asian)” instead of the incorrect “Chinese (European)”. The correct version of the 14^th^ row of the first column states “Japanese (East Asian)” instead of the incorrect “Japanese (European)”. This has been corrected in both the PDF and HTML versions of the Article.

