# Supplementary material for: Genetic regulation of serum IgA levels and susceptibility to common immune, infectious, kidney, and cardio-metabolic traits
Source: Nat Commun. 2022 Nov 11;13:6859. doi: 10.1038/s41467-022-34456-6 (PMC9651905; doi:10.1038/s41467-022-34456-6)
Supplement: Supplementary file 1 — Supplementary Information [file 41467_2022_34456_MOESM1_ESM.pdf]

SUPPLEMENTAL INFORMATION:

Genetic regulation of serum IgA levels and susceptibility to common immune, infectious, kidney, and cardio-metabolic traits

Lili Liu et al.

Table of Contents

SUPPLEMENTAL INFORMATION: *1*

SUPPLEMENTAL FIGURES *3*

Supplementary Figure 1. 3

Supplementary Figure 2. 4

Supplementary Figure 3. 5

Supplementary Figure 4. 6

Supplementary Figure 5. 7

Supplementary Figure 6. 8

Supplementary Figure 7. 9

Supplementary Figure 8. 10

SUPPLEMENTAL TABLES *11*

Supplementary Table 1. 11

Supplementary Table 2. 12

Supplementary Table 3. 13

Supplementary Table 4. 14

Supplementary Table 5. 15

Supplementary Table 6. 16

Supplementary Table 7. 17

Supplementary Table 8. 18

Supplementary Table 9. 19

Supplementary Table 10. 20

Supplementary Table 11. 21

Supplementary Table 12. 22

Supplementary Table 13. 23

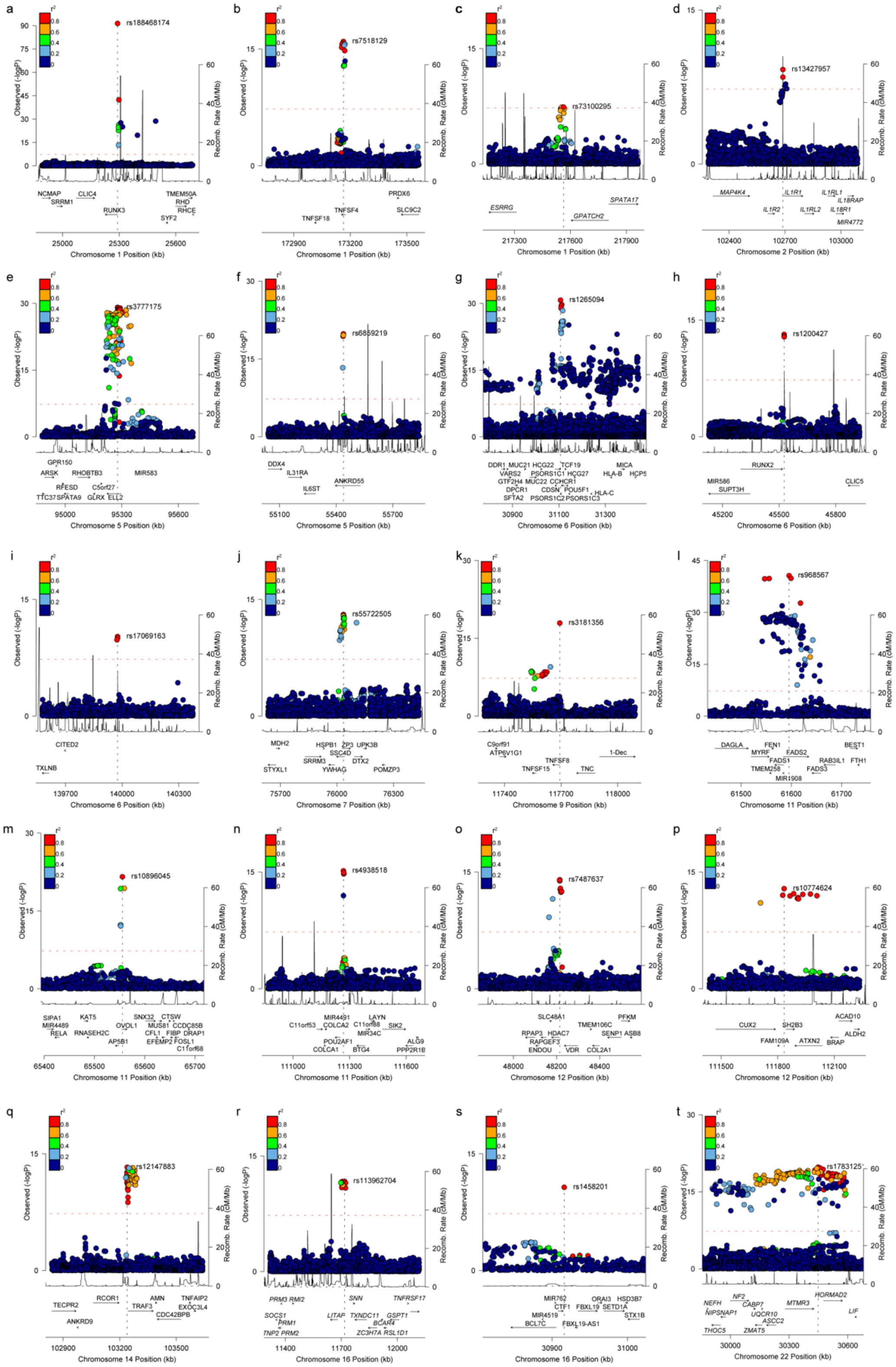

Supplementary Figure 1. Regional plots for 20 genome-wide significant loci: (a) *RUNX3*, **(b)** *TNFSF4-TNFSF18*, **(c)** *GPATCH2*, **(d)** *IL1R1*, **(e)** *ELL2*, **(f)** *ANKRD55-IL6ST*, **(g)** *HLA*, **(h)** *RUNX2*, **(i)** *CITED2*, **(j)** *ZP3-SSC4D*, **(k)** *TNFSF8-TNFSF15*, **(l)** *FADS2-TMEM258*, **(m)** *OVOL1-RELA*, **(n)** *POU2AF1*, **(o)** *HDAC7*, **(p)** *SH2B3*, **(q)** *TRAF3*, **(r)** *LITAF*, **(s)** *CTF1*, and **(t)** *HORMAD2-LIF* loci. The x-axis shows the physical position in Mb (hg19 coordinates) along with known genes in the region; the left y-axis presents -log10 two-sided p-values (fixed effects GWAS meta-analysis for serum IgA levels) and the right y-axis shows the recombination rate across the region (1000 Genomes, all populations); the dotted horizontal line indicates a genome-wide significant threshold of 5.0E-08.

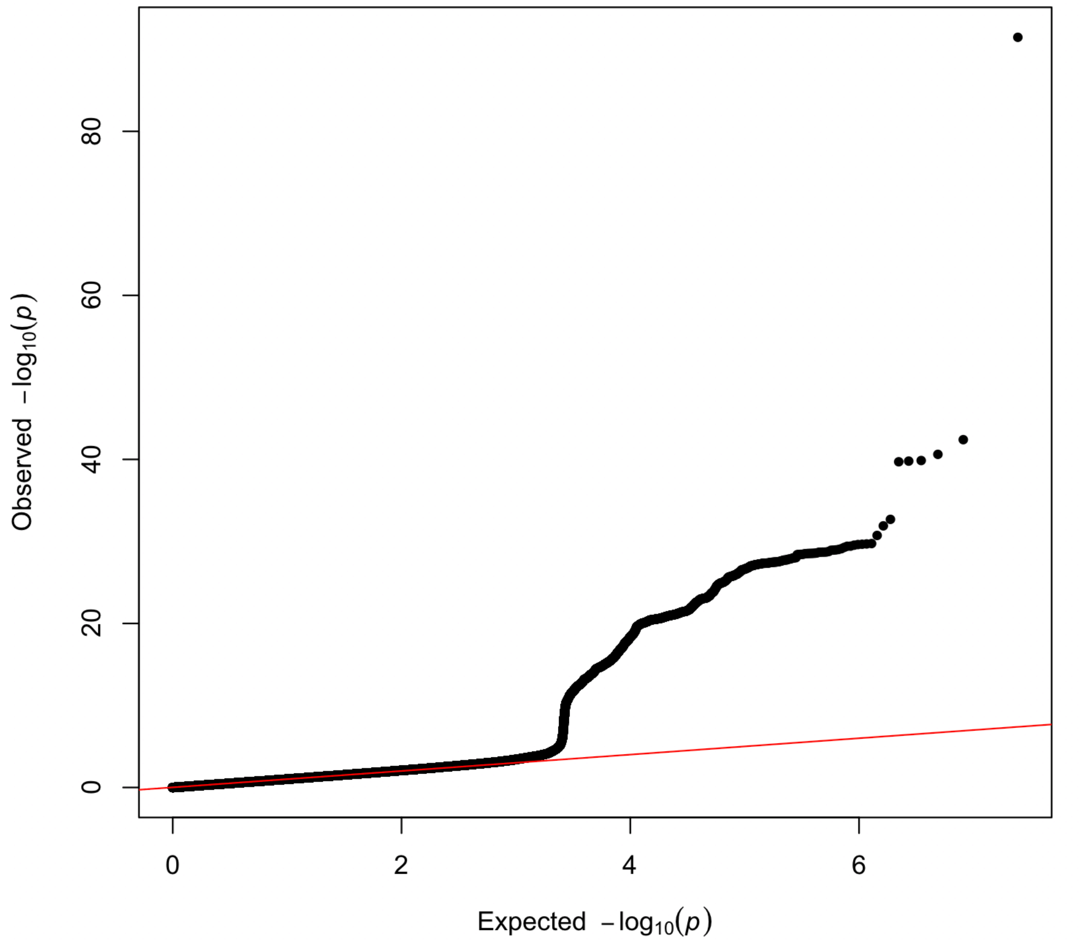

Supplementary Figure 2. Quantile-quantile (QQ) plot of the trans-ethnic meta-analysis (total N = 41,263 individuals). The observed -log10 P-values (y-axis) correspond to the two-sided tests of individual variants in genome-wide fixed effects meta-analysis. The x-axis depicts the expected two-sided P-values under the null hypothesis. The overall meta-analysis genomic inflation factor (λ) was estimated at 1.016.

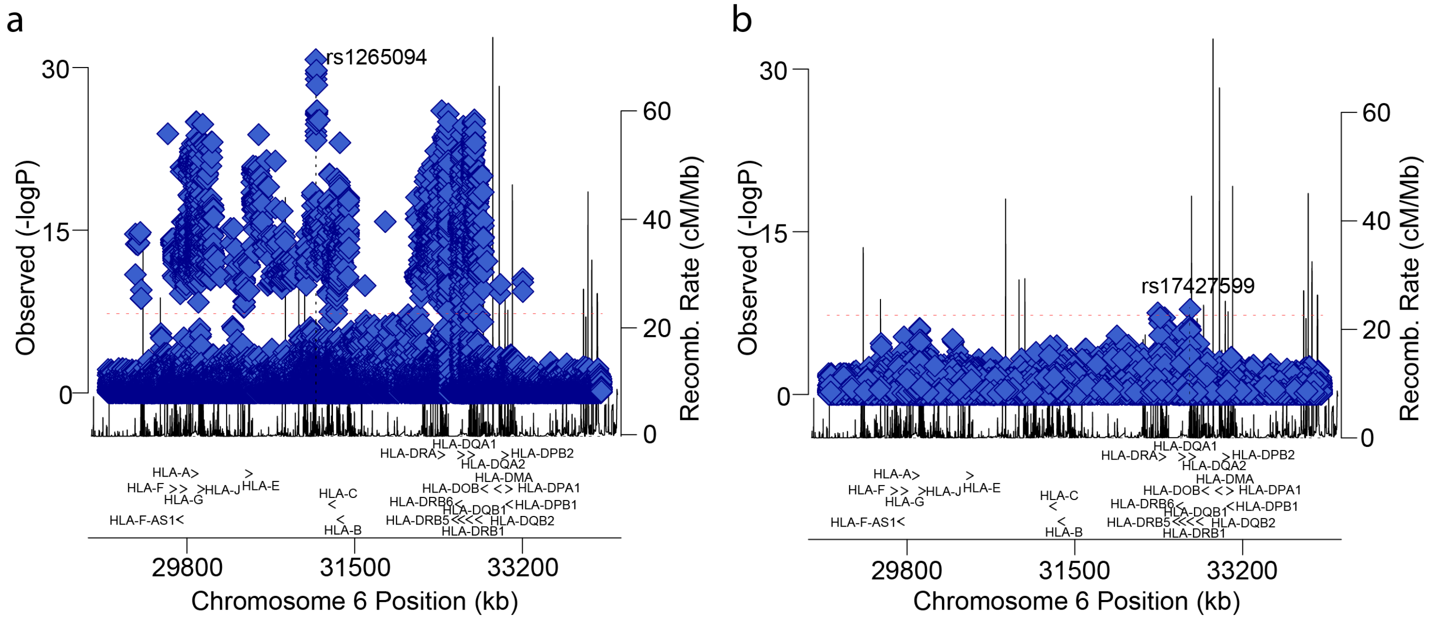

Supplementary Figure 3. Stepwise conditional analysis of the HLA locus. (a) Regional plot for the fixed-effects meta-analysis without conditioning; rs1265094 represents the top SNP; **(b)** Regional plot for the meta-analysis after conditioning on rs1265094 depicting independent genome-wide significant signal with rs17427599 as the top SNP; the left y-axis presents -log10 two-sided p-values for association statistics (fixed effects meta-analysis) and the right y-axis shows the recombination rate across the region (1000 Genomes, all populations); the dotted horizontal line indicates a genome-wide significant threshold of 5.0E-08; gene annotations are limited only to HLA genes for positional reference.

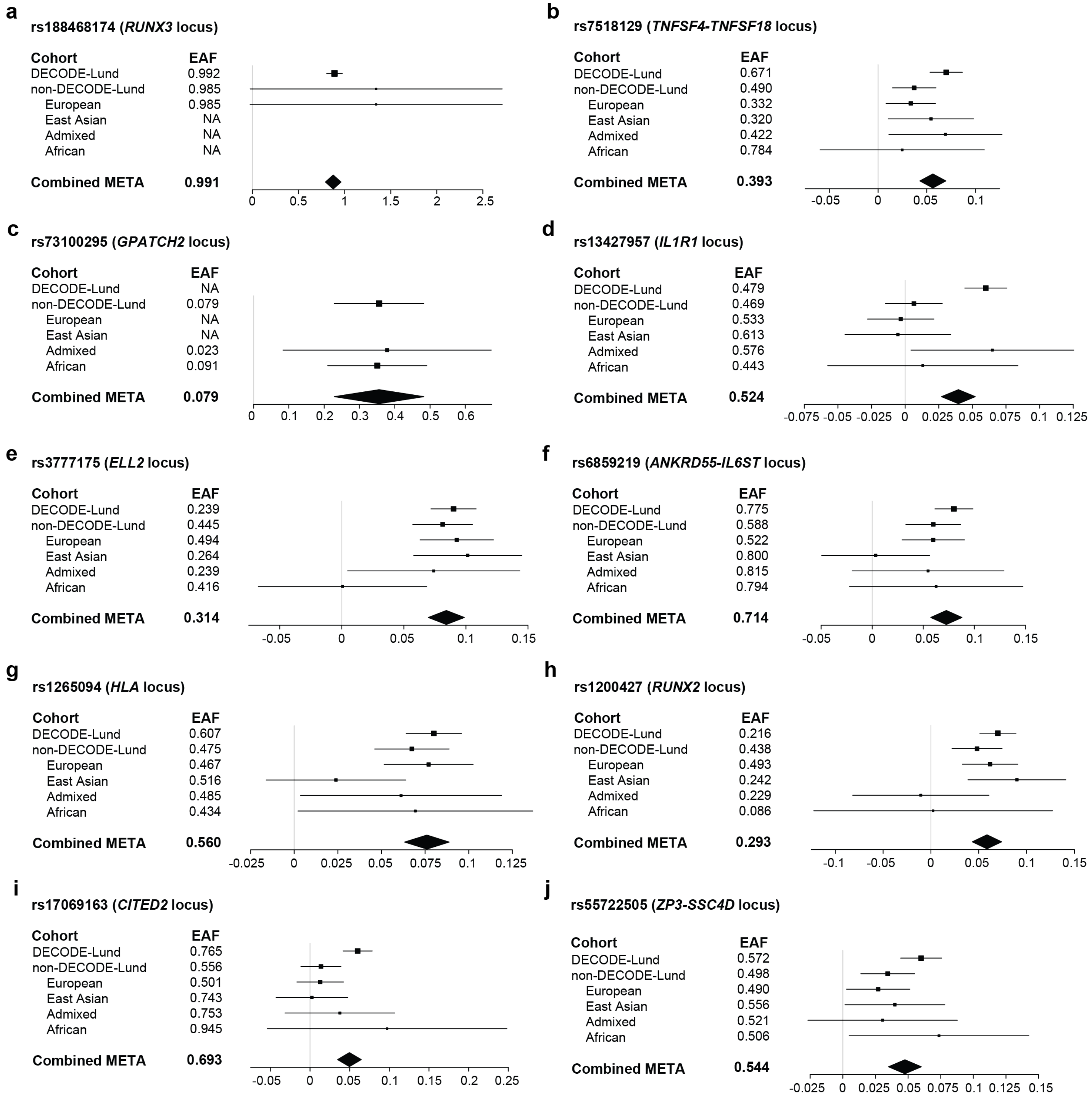

Supplementary Figure 4. Forest plots with effect estimates (and 95% confidence intervals) for the deCODE-Lund and non-deCODE-Lund cohorts, and by ancestry, for the first 10 genome-wide significant loci ordered from a to j by chromosome and genomic coordinates. EAF: Effect Allele Frequency, averaged for included cohorts. The center of each bar corresponds to the effect estimate (beta), the error bars correspond to 95% confidence intervals for the effect size. Decode-Lund N= 19,034; non-Decode-Lund N=22,229; European N=35,094 ; East Asian N=1,957 ; Admixed N=2,461; African N=1,751.

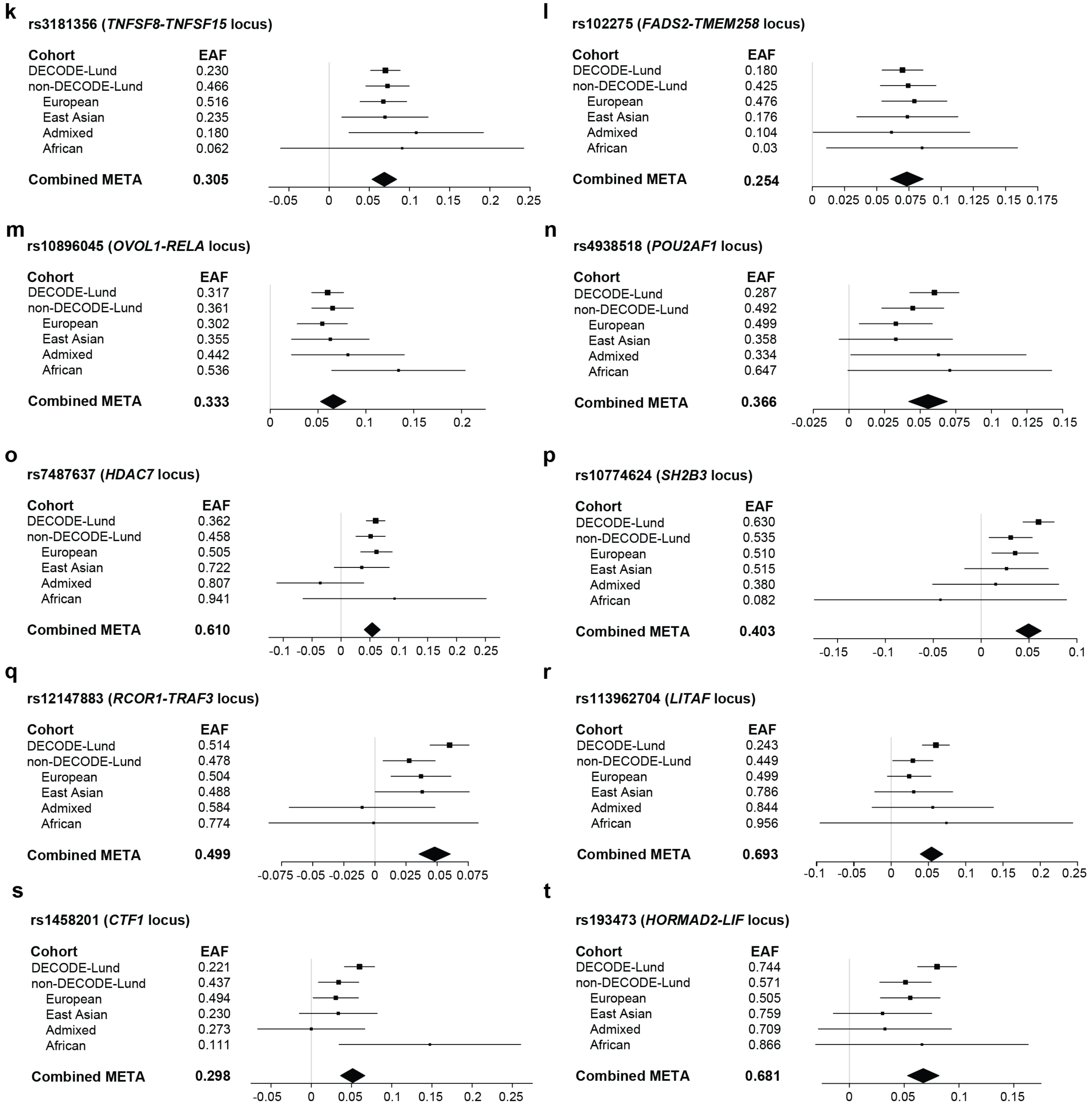

Supplementary Figure 5. Forest plots with effect estimates (and 95% confidence intervals) for the deCODE-Lund and non-deCODE-Lund cohorts, and by ancestry, for the last 10 genome-wide significant loci ordered from k to t by chromosome and genomic coordinates. EAF: Effect Allele Frequency, averaged for included cohorts. The center of each bar corresponds to the effect estimate (beta), the error bars correspond to 95% confidence intervals for the effect size. Decode-Lund N= 19,034; non-Decode-Lund N=22,229; European N=35,094 ; East Asian N=1,957 ; Admixed N=2,461; African N=1,751.

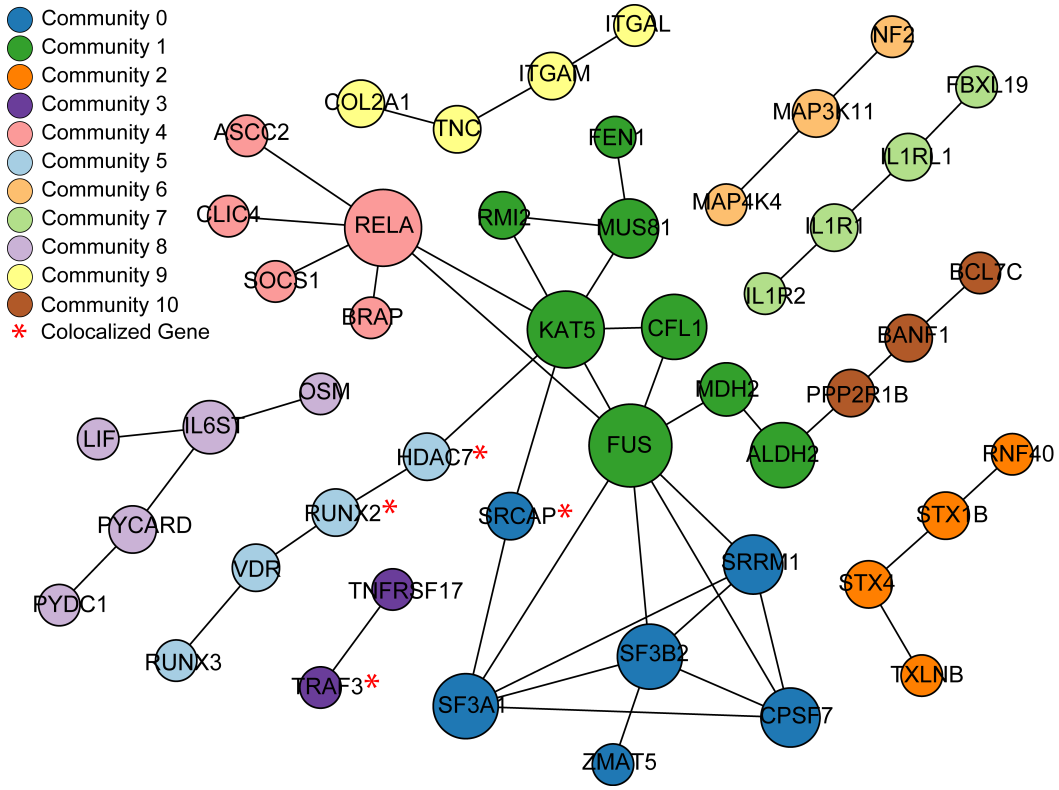

Supplementary Figure 6. Protein-protein interaction (PPI) network for positional candidate genes at GWAS loci: each color represents a distinct network module (a group of inter-connected genes); red asterisk indicates e-genes co-localized with GWAS loci. Overall, this PPI network has more connectivity than expected by chance (permutation P=2e-03). The PPI information was obtained from InWeb_IM.

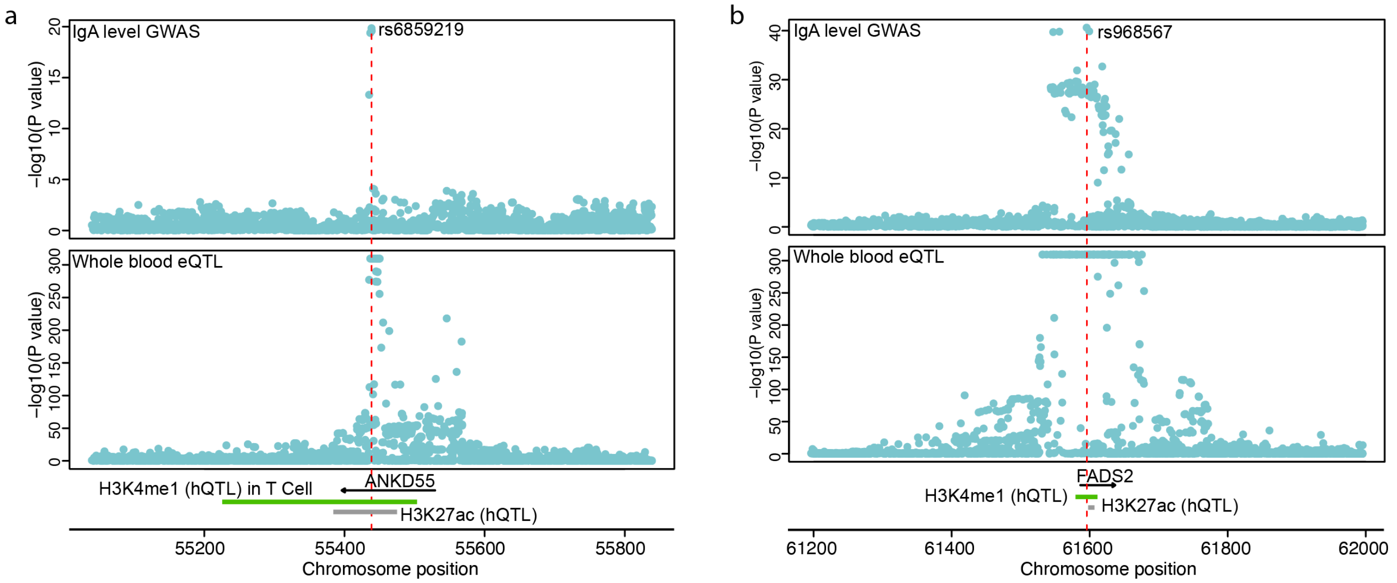

Supplementary Figure 7. Integrative analysis of eQTL and hQTL for a) *ANKRD55* and b) *FADS1/FDAS2* loci. The upper and lower panels show the regional plots for IgA GWAS and eQTLs, respectively. The y-axis represents the -log10 of the two-sided p-value (upper panel: fixed effects GWAS meta-analysis for IgA levels; lower panel: Wald test from linear regression of individual variants against gene expression levels) and x-axis shows the chromosome positions. The positions of candidate genes and hQTL peaks are depicted above the x-axis.

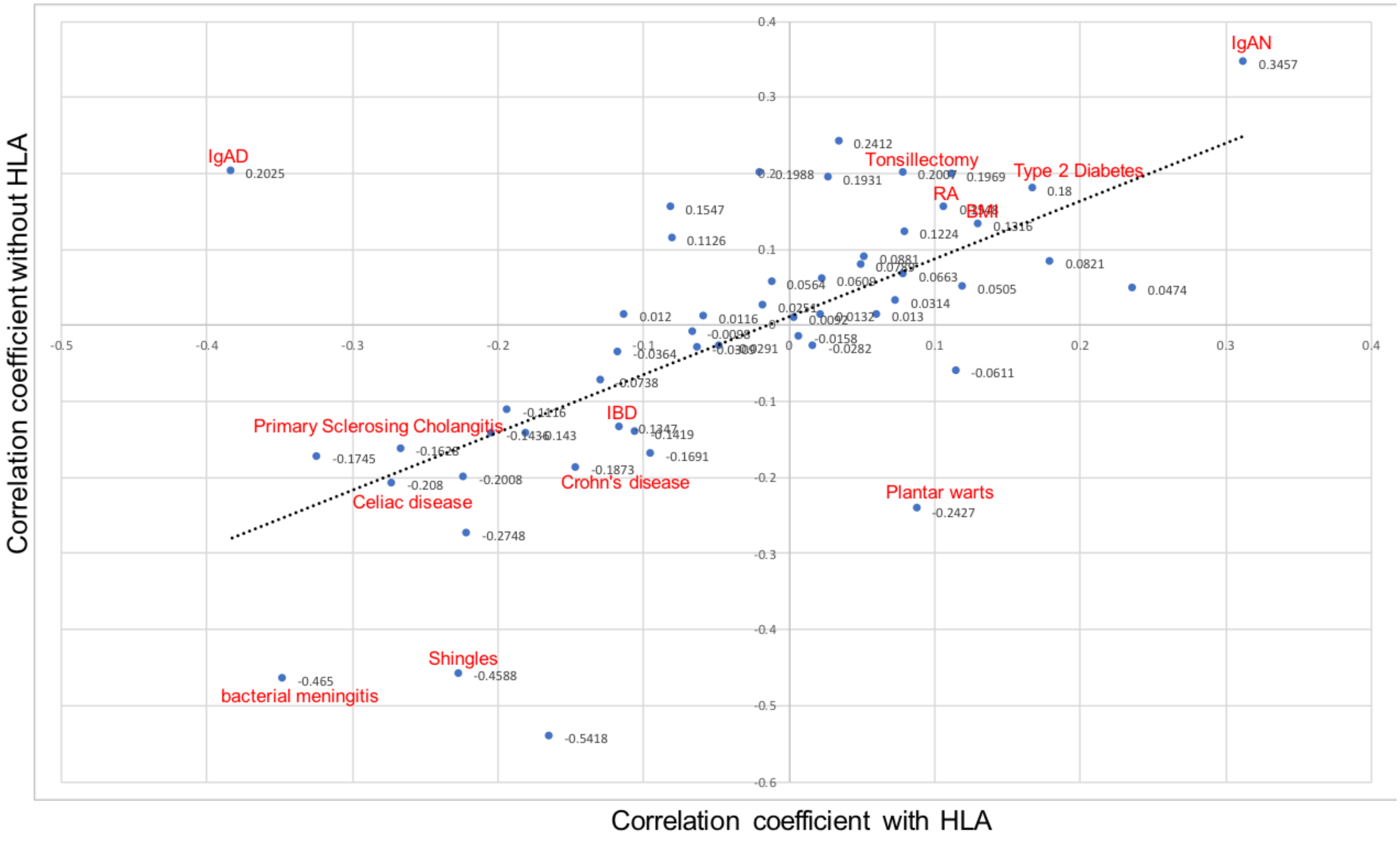

Supplementary Figure 8. Genome-wide genetic correlations between serum IgA level and other complex traits with HLA region (x-axis) and without HLA region (y-axis). Nominally significant correlations (genome-wide genetic correlation by LDSC, two-sided $p<0.05$) are labeled in red. For two traits, IgA Deficiency (IgAD) and Plantar Warts, the inclusion of HLA region flips the sign of the genome-wide genetic correlation coefficient with serum IgA levels. IgAN: IgA nephropathy; IBD: Inflammatory Bowel Disease; RA: Rheumatoid Arthritis; BMI: Body Mass Index.

Supplementary Table 1. Suggestive loci in the combined trans-ethnic meta-analysis (P<1.0E-06). P-values: two-sided fixed effects meta-analysis. Gene names indicated in italics.

Locus	CHR	BP (hg19)	SNP	Risk Allele	BETA	P-value	Note
TNFSF13	17	7462969	rs3803800	A	0.06	9.41E-08	Known
DLEU1	13	50981003	rs113429750	C	0.17	2.86E-07	Novel
GUCY1A2	11	106739921	rs10431056	C	0.07	3.21E-07	Novel
TNNT1	19	55658222	rs11550309	T	0.97	5.01E-07	Novel
ARHGEF17	11	73080563	rs115997298	G	0.50	5.38E-07	Novel
DZANK1, ZNF133	20	18343798	rs6045361	G	0.05	7.73E-07	Novel
DCLK2	4	150953126	rs77297307	C	0.46	8.21E-07	Novel
PDE10A	6	166396027	rs73260573	G	0.07	9.86E-07	Novel

Supplementary Table 2. Pathway enrichment analysis: top pathways enriched in genes encoded by the significant GWAS loci for serum IgA levels; P-value: unadjusted two-sided enrichment test; B&H: Benjamini and Hochberg adjustment; B&Y: Benjamini and Yekuteli adjustment.

Name	Source	P-value	FDR B&H	FDR B&Y	Bonferroni	No. of Genes (input)	No. of Genes (annotation)
Cytokine Signaling in Immune system	REACTOME	1.17E-06	1.08E-03	7.99E-03	1.08E-03	24	763
Cytokine-cytokine receptor interaction	KEGG	5.23E-06	2.41E-03	1.79E-02	4.82E-03	13	270
Signaling by Interleukins	REACTOME	1.10E-05	3.18E-03	2.36E-02	1.01E-02	18	531
TNFs bind their physiological receptors	REACTOME	1.38E-05	3.18E-03	2.36E-02	1.27E-02	5	30
IL-6-type cytokine receptor ligand interactions	REACTOME	4.98E-05	9.19E-03	6.80E-02	4.59E-02	4	20
Linoleic acid (LA) metabolism	REACTOME	1.29E-04	1.99E-02	1.48E-01	1.20E-01	3	10
Interleukin-6 family signaling	REACTOME	1.70E-04	2.25E-02	1.66E-01	1.57E-01	4	27
IL12-mediated signaling events	Pathway Interaction Database	4.43E-04	3.96E-02	2.93E-01	4.09E-01	5	61
alpha-linolenic acid (ALA) metabolism	REACTOME	4.72E-04	3.96E-02	2.93E-01	4.36E-01	3	15
TACI and BCMA stimulation of B cell immune responses.	MSigDB C2 BIOCARTA	4.72E-04	3.96E-02	2.93E-01	4.36E-01	3	15

Supplementary Table 3. Top ranked tissues and cell types based on DEPICT analysis. The analysis was performed for 209 Medical Subject Heading (MeSH) tissue and cell type annotations, only results at nominal P<0.05 (two-sided unadjusted enrichment test) are displayed and sorted by the level of statistical significance, the results meeting FDR <5% highlighted in red (and graphically depicted in Figure 2b).

MeSH term	MeSH first level term	MeSH second level term	MeSH first level term	Nominal P value	FDR < 5%
A15.378.316	Bone Marrow Cells	Hemic and Immune Systems	Bone Marrow Cells	4.89E-05	Yes
A15.378	Hematopoietic System	Hemic and Immune Systems	Hematopoietic System	4.89E-05	Yes
A15.145	Blood	Hemic and Immune Systems	Blood	2.77E-04	Yes
A11.627	Myeloid Cells	Cells	Myeloid Cells	2.79E-04	Yes
A15.382.680	Phagocytes	Hemic and Immune Systems	Phagocytes	3.31E-04	Yes
A15.145.229	Blood Cells	Hemic and Immune Systems	Blood Cells	4.20E-04	Yes
A15.145.300	Fetal Blood	Hemic and Immune Systems	Fetal Blood	6.36E-04	Yes
A15.382.490.315.583	Neutrophils	Hemic and Immune Systems	Neutrophils	6.39E-04	Yes
A11.118.637.415	Granulocytes	Cells	Granulocytes	6.57E-04	Yes
A11.118.637	Leukocytes	Cells	Leukocytes	8.90E-04	Yes
A02.835.583.443.800.800	Synovial Fluid	Musculoskeletal System	Synovial Fluid	0.0016	Yes
A15.378.316.580	Monocytes	Hemic and Immune Systems	Monocytes	0.0019	Yes
A15.382	Immune System	Hemic and Immune Systems	Immune System	0.003	Yes
A11.066	Antigen Presenting Cells	Cells	Antigen Presenting Cells	0.003	Yes
A15.382.812.260	Dendritic Cells	Hemic and Immune Systems	Dendritic Cells	0.003	Yes
A15.382.520.604.700	Spleen	Hemic and Immune Systems	Spleen	0.004	Yes
A15.382.812	Mononuclear Phagocyte System	Hemic and Immune Systems	Mononuclear Phagocyte System	0.005	Yes
A15.382.520	Lymphatic System	Hemic and Immune Systems	Lymphatic System	0.008	No
A10.549	Lymphoid Tissue	Tissues	Lymphoid Tissue	0.008	No
A10.549.400	Lymph Nodes	Tissues	Lymph Nodes	0.009	No
A02.835.232.043	Bones of Lower Extremity	Musculoskeletal System	Bones of Lower Extremity	0.010	No
A15.145.229.637.555	Leukocytes Mononuclear	Hemic and Immune Systems	Leukocytes Mononuclear	0.012	No
A11.118.637.555.567.569	T Lymphocytes	Cells	T Lymphocytes	0.012	No
A02.835.232.043.300	Foot Bones	Musculoskeletal System	Foot Bones	0.013	No
A02.835.232.043.300.710	Tarsal Bones	Musculoskeletal System	Tarsal Bones	0.013	No
A11.118.637.555.567.569.200.700	T Lymphocytes Regulatory	Cells	T Lymphocytes Regulatory	0.016	No
A09.371.060	Anterior Eye Segment	Sense Organs	Anterior Eye Segment	0.018	No
A09.371.337.168	Conjunctiva	Sense Organs	Conjunctiva	0.018	No
A09.371.337	Eyelids	Sense Organs	Eyelids	0.018	No
A15.145.229.637.555.567.569.200	CD4 Positive T Lymphocytes	Hemic and Immune Systems	CD4 Positive T Lymphocytes	0.020	No
A15.382.490.555.567.537	Killer Cells Natural	Hemic and Immune Systems	Killer Cells Natural	0.020	No
A10.165	Connective Tissue	Tissues	Connective Tissue	0.020	No
A15.382.216	Bone Marrow	Hemic and Immune Systems	Bone Marrow	0.021	No
A02.835	Skeleton	Musculoskeletal System	Skeleton	0.024	No
A02.835.232	Bone and Bones	Musculoskeletal System	Bone and Bones	0.024	No
A15.382.812.522	Macrophages	Hemic and Immune Systems	Macrophages	0.029	No
A11.872.378.294	Lymphoid Progenitor Cells	Cells	Lymphoid Progenitor Cells	0.032	No
A11.118.637.555.567.562.440	Precursor Cells B Lymphoid	Cells	Precursor Cells B Lymphoid	0.032	No
A04.531.520	Nasal Mucosa	Respiratory System	Nasal Mucosa	0.032	No
A09.531	Nose	Sense Organs	Nose	0.032	No
A10.615.550.760	Respiratory Mucosa	Tissues	Respiratory Mucosa	0.032	No
A11.872.378	Hematopoietic Stem Cells	Cells	Hematopoietic Stem Cells	0.042	No

Supplementary Table 4. ANNOVAR annotation of the top significant SNPs and their proxies (R2>0.5). * indicates top SNP in GWAS. P-values (discovery): two-sided fixed effects meta-analysis. Gene names indicated in italics.

Locus	SNP	Chr.	Position (hg19)	Risk allele	Non-risk allele	Beta	P (discovery)	R^2 with top SNP	Annotation
OVOL1	rs10896045	11	65555524	**a**	g	0.066	2.57E-22	1.00*	intronic
POU2AF1	rs4938518	11	111267394	t	c	0.056	7.01E-16	1.00*	intergenic
HDAC7	rs7487637	12	48214825	a	g	-0.054	9.97E-15	1.00*	intergenic
RCOR1,TRAF3	rs12147883	14	103239630	t	c	-0.048	5.42E-14	1.00*	intergenic b/w TRAF3 and RCOR1
RCOR1,TRAF3	rs1051750	14	103243946	t	c	-0.045	4.72E-11	0.82	UTR5 of TRAF3
RUNX2	rs1200427	6	45526470	a	t	-0.059	6.85E-14	1.00*	intergenic b/w RUNX2 and CLIC5
RUNX2	rs1200428	6	45518202	t	g	0.035	8.47E-03	0.51	UTR3 of RUNX2
ZP3,SSC4D	rs55722505	7	76034150	c	g	0.048	8.61E-14	1.00*	intronic
ZP3,SSC4D	rs10261314	7	76023029	t	c	0.044	3.95E-12	0.62	synonymous in SSC4D
LITAF	rs113962704	16	11717832	a	t	-0.054	1.91E-12	1.00*	intergenic b/w LITAF and SNN
CTF1	rs1458201	16	30916129	a	g	0.052	2.02E-11	1.00*	intergenic b/w CTF1 and FBXL19-AS1
CTF1	rs35675346	16	30936081	a	g	0.028	0.02305	0.57	missense in FBXL19
FILNC1	rs17069163	6	139975943	t	c	0.050	5.94E-11	1.00*	intergenic b/w LINC01625 and LOC100132735
IL1R1	rs13427957	2	102689031	t	c	-0.040	6.19E-10	1.00*	intronic
RUNX3	rs188468174	1	25291697	t	c	-0.876	3.42E-92	1.00*	intergenic b/w RUNX3 and MIR4425
FADS2	rs968567	11	61595564	t	c	0.116	2.42E-41	1.00*	UTR5 of FADS2
ELL2	rs3777175	5	95277555	a	g	-0.084	7.81E-30	1.00*	intergenic
ELL2	rs3777203	5	95234377	t	c	0.076	9.20E-28	0.74	synonymous in ELL2
ELL2	rs17085249	5	95236415	a	g	0.076	8.59E-28	0.70	synonymous in ELL2
ELL2	rs3815768	5	95236459	t	c	0.076	2.91E-27	0.69	missense in ELL2 (BENIGN, 0.0)
ELL2	rs3777202	5	95234392	a	c	-0.076	5.22E-28	0.67	synonymous in ELL2
ELL2	rs17085231	5	95222156	t	c	0.077	4.94E-28	0.65	UTR3 of ELL2
ELL2	rs11135442	5	95222511	t	c	-0.077	1.31E-28	0.60	UTR3 of ELL2
ELL2	rs3777204	5	95234350	a	g	-0.074	1.23E-26	0.56	synonymous in ELL2
ELL2	rs1043381	5	95221337	t	c	0.073	5.44E-26	0.50	UTR3 of ELL2
ANKRD55	rs6859219	5	55438580	a	c	-0.073	1.41E-20	1.00*	intronic
HORMAD2	rs193473	22	30448399	a	g	0.067	1.58E-20	1.00*	intronic
TNFSF8	rs3181356	9	117692882	t	c	0.069	1.13E-18	1.00	upstream TNFSF8 (dist=7)
TNFSF8	rs55918341	9	117547198	ca	c	-0.026	0.3385	0.68	UTR3 of TNFSF15
TNFSF8	rs7853287	9	117549327	a	g	0.078	3.95E-09	0.68	UTR3 of TNFSF15
TNFSF4	rs7518129	1	173163568	a	g	-0.056	1.06E-16	1.00*	intergenic
TNFSF4	rs7514229	1	173154304	t	g	0.055	6.13E-16	0.83	UTR3 of TNFSF4
SH2B3	rs10774624	12	111833788	a	g	-0.050	1.37E-13	1.00*	intergenic b/w FAM109A and SH2B3
SH2B3	rs3184504	12	111884608	t	c	0.048	6.07E-13	0.94	missense in SH2B3 (BENIGN, 0.0)

Supplementary Table 5. Colocalization analysis with eQTLs from whole blood and 13 primary immune cell types. Posterior probabilities of sharing and not sharing the same causal variant between GWAS and eQTL are designated as PP4 and PP3, respectively. The designation of a "concordant direction" indicates that IgA level increasing allele was also associated with higher expression of corresponding genes; "opposite direction" indicates that the IgA increasing allele was associated with lower expression of corresponding genes. GWAS P-values: two-sided fixed effects meta-analysis. eSNP P-value: Wald test from linear regression of individual variants against gene expression levels. Gene names indicated in italics.

Cell type	Chr	GWAS Locus Region (Start-End)	GWAS Lead SNP	GWAS p Value	eSNP	eSNP p Value	PP3	PP4	Gene	Direction
B cell NAÏVE	6	30706942-31506854	rs1265094	1.86E-31	rs720467	2.95E-07	0.34	0.56	*TCF19*	Opposite
	7	75634687-76433936	rs55722505	8.61E-14	rs201874621	9.30E-09	0.34	0.58	*POMZP3*	Concordant
	11	61196289-61995513	rs968567	2.42E-41	rs174528	1.70E-11	0.16	0.84	*FADS1*	Concordant
	11	61196289-61995513	rs968567	2.42E-41	rs7943728	2.18E-20	0.00	1.00	*FADS2*	Concordant
CD4 NAÏVE	11	61196289-61995513	rs968567	2.42E-41	rs174576	5.65E-18	0.23	0.77	*FADS1*	Concordant
	11	61196289-61995513	rs968567	2.42E-41	rs7943728	7.33E-16	0.00	1.00	*FADS2*	Concordant
	14	102840084-103639456	rs12147883	5.42E-14	rs4906263	9.77E-05	0.07	0.61	*TRAF3*	Concordant
CD4 STIM	11	61196289-61995513	rs968567	2.42E-41	rs7943728	5.22E-13	0.01	0.99	*FADS2*	Concordant
CD8 NAÏVE	6	30706942-31506854	rs1265094	1.86E-31	rs2535324	1.84E-04	0.01	0.55	*HCG4*	Concordant
	7	75634687-76433936	rs55722505	8.61E-14	rs201874621	3.86E-11	0.41	0.58	*POMZP3*	Concordant
	11	61196289-61995513	rs968567	2.42E-41	rs7943728	5.61E-16	0.02	0.98	*FADS1*	Concordant
	11	61196289-61995513	rs968567	2.42E-41	rs7943728	9.88E-16	0.00	1.00	*FADS2*	Concordant
	14	102840084-103639456	rs12147883	5.42E-14	rs7158166	1.13E-06	0.07	0.78	*TRAF3*	Concordant
CD8 STIM	6	30706942-31506854	rs1265094	1.86E-31	rs2535324	8.93E-05	0.01	0.63	*HCG4*	Concordant
	11	61196289-61995513	rs968567	2.42E-41	rs174544	1.83E-16	0.31	0.69	*FADS1*	Concordant
	11	61196289-61995513	rs968567	2.42E-41	rs7943728	1.53E-14	0.00	1.00	*FADS2*	Concordant
M2	11	61196289-61995513	rs968567	2.42E-41	rs174538	4.33E-14	0.10	0.90	*FADS2*	Concordant
	16	11317993-12117637	rs113962704	1.91E-12	rs9932684	3.77E-05	0.11	0.52	*AC099489.1*	Concordant
	16	11317993-12117637	rs113962704	1.91E-12	rs9932684	8.75E-07	0.06	0.81	*LITAF*	Concordant
Monocytes	7	75634687-76433936	rs55722505	8.61E-14	rs1799237	4.20E-11	0.31	0.69	*ZP3*	Opposite
	7	75634687-76433936	rs55722505	8.61E-14	rs4728708	2.59E-12	0.22	0.78	*SSC4D*	Opposite
	11	61196289-61995513	rs968567	2.42E-41	rs7943728	1.46E-14	0.00	1.00	*FADS2*	Concordant
	16	11317993-12117637	rs113962704	1.91E-12	rs72781049	1.73E-12	0.02	0.98	*LITAF*	Concordant
NK	11	61196289-61995513	rs968567	2.42E-41	rs7943728	3.87E-14	0.03	0.97	*FADS2*	Concordant
TFH	11	61196289-61995513	rs968567	2.42E-41	rs174574	1.69E-06	0.13	0.67	*TMEM258*	Concordant
	11	61196289-61995513	rs968567	2.42E-41	rs7943728	3.96E-19	0.00	1.00	*FADS2*	Concordant
	14	102840084-103639456	rs12147883	5.42E-14	rs2075771	1.71E-05	0.07	0.71	*TRAF3*	Concordant
TH1	2	102289183-103088777	rs13427957	6.19E-10	rs13401717	1.06E-04	0.05	0.69	*MIR4772*	Concordant
	11	61196289-61995513	rs968567	2.42E-41	rs174544	1.74E-13	0.26	0.74	*FADS1*	Concordant
	11	61196289-61995513	rs968567	2.42E-41	rs7943728	2.250e-17	0.00	1.00	*FADS2*	Concordant
	12	47814851-48614567	rs7487637	9.97E-15	rs11168256	1.57E-04	0.08	0.59	*HDAC7*	Concordant
	14	102840084-103639456	rs12147883	5.42E-14	rs2075771	1.88E-05	0.08	0.58	*TRAF3*	Concordant
TH2	1	172763639-173563418	rs7518129	1.06E-16	rs59597894	6.26E-05	0.09	0.50	*TNFSF18*	Opposite
	11	61196289-61995513	rs968567	2.416E-41	rs796587134	2.14E-07	0.24	0.51	*MYRF*	Concordant
	11	61196289-61995513	rs968567	2.416E-41	rs7943728	2.02E-06	0.03	0.89	*TMEM258*	Concordant
	11	61196289-61995513	rs968567	2.416E-41	rs174544	5.90E-21	0.00	1.00	*FADS2*	Concordant
	14	102840084-103639456	rs12147883	5.42E-14	rs12878532	5.02E-06	0.06	0.84	*TRAF3*	Concordant
TH17	11	61196289-61995513	rs968567	2.416E-41	rs174528	3.83E-06	0.09	0.71	*TMEM258*	Concordant
	11	61196289-61995513	rs968567	2.416E-41	rs7943728	7.17E-20	0.00	1.00	*FADS2*	Concordant
	14	102840084-103639456	rs12147883	5.416E-14	rs79589176	6.04E-05	0.09	0.57	*TRAF3*	Concordant
	22	30048571-30848388	rs193473	1.58E-20	rs249398	3.58E-05	0.15	0.52	*CNN2P1*	Opposite
THSTAR	6	30706942-31506854	rs1265094	1.858E-31	rs3130702	3.49E-05	0.27	0.55	*APOM*	Concordant
	11	61196289-61995513	rs968567	2.416E-41	rs174576	3.63E-17	0.36	0.64	*FADS1*	Concordant
	11	61196289-61995513	rs968567	2.416E-41	rs7943728	1.32E-14	0.01	0.99	*FADS2*	Concordant
	14	102840084-103639456	rs12147883	5.416E-14	rs2075771	4.04E-06	0.07	0.76	*TRAF3*	Concordant
	22	30048571-30848388	rs193473	1.58E-20	rs199726005	3.38E-05	0.11	0.61	*RF00019*	Concordant
TREG MEM	5	55039671-55838571	rs6859219	1.413E-20	rs7731626	3.02E-08	0.12	0.51	*ANKRD55*	Concordant
	6	30706942-31506854	rs1265094	1.858E-31	rs1265157	1.01E-04	0.18	0.60	*PRRT1*	Opposite
	11	61196289-61995513	rs968567	2.416E-41	rs174576	2.69E-18	0.22	0.78	*FADS1*	Concordant
	11	61196289-61995513	rs968567	2.416E-41	rs7943728	6.73E-18	0.00	1.00	*FADS2*	Concordant
	11	61196289-61995513	rs968567	2.416E-41	rs174585	1.64E-10	0.15	0.83	*FADS3*	Concordant
	14	102840084-103639456	rs12147883	5.416E-14	rs12887521	8.63E-06	0.08	0.76	*TRAF3*	Concordant
	16	30516148-31314599	rs1458201	2.017E-11	rs11865499	1.58E-05	0.05	0.67	*KAT8*	Concordant
TREG NAIVE	11	61196289-61995513	rs968567	2.416E-41	rs7943728	4.97E-18	0.00	1.00	*FADS1*	Concordant
	11	61196289-61995513	rs968567	2.416E-41	rs7943728	1.83E-15	0.00	1.00	*FADS2*	Concordant
Whole blood	5	55039671-55838571	rs6859219	1.413E-20	rs7731626	3.27E-310	0.02	0.98	*ANKRD55*	Concordant
	5	94878263-95677384	rs3777175	7.813E-30	rs1841010	1.16E-37	0.47	0.53	*ELL2*	Opposite
	6	30706942-31506854	rs1265094	1.858E-31	rs114986457	9.23E-25	0.00	1.00	*HCG4*	Concordant
	6	30706942-31506854	rs1265094	1.858E-31	rs145930603	2.45E-35	0.02	0.98	*HCG4P3*	Opposite
	6	30706942-31506854	rs1265094	1.858E-31	rs116481148	3.27E-310	0.04	0.93	*HLA-DRB5*	Concordant
	6	30706942-31506854	rs1265094	1.858E-31	rs116239366	1.27E-20	0.07	0.93	*DDX39BP2*	Opposite
	6	30706942-31506854	rs1265094	1.858E-31	rs114986457	1.33E-101	0.47	0.53	*HLA-K*	Concordant
	6	45127152-45926460	rs1200427	6.853E-14	rs1200428	3.27E-310	0.01	0.99	*RUNX2*	Opposite
	7	75634687-76433936	rs55722505	8.611E-14	rs55650029	5.74E-42	0.04	0.96	*SRCRB4D*	Opposite
	7	75634687-76433936	rs55722505	8.611E-14	rs6966715	3.27E-310	0.14	0.86	*DTX2*	Opposite
	7	75634687-76433936	rs55722505	8.611E-14	rs4415243	3.47E-227	0.47	0.53	*YWHAG*	Opposite
	11	61196289-61995513	rs968567	2.416E-41	rs61896141	3.27E-310	0.09	0.91	*FADS1*	Concordant
	11	61196289-61995513	rs968567	2.416E-41	rs968567	3.27E-310	0.15	0.85	*FADS2*	Concordant
	12	111437618-112233495	rs10774624	1.374E-13	rs597808	6.93E-07	0.01	0.99	*PPTC7*	Opposite
	12	111437618-112233495	rs10774624	1.374E-13	rs138281696	2.47E-45	0.02	0.98	*TRAFD1*	Concordant
	12	111437618-112233495	rs10774624	1.374E-13	rs73197952	1.07E-09	0.02	0.97	*HVCN1*	Opposite
	16	30516148-31314599	rs1458201	2.017E-11	rs4889653	3.00E-06	0.09	0.89	*SRCAP*	Concordant
	16	30516148-31314599	rs1458201	2.017E-11	rs17855121	1.05E-07	0.14	0.86	*PPP4C*	Opposite
	16	30516148-31314599	rs1458201	2.017E-11	rs4346218	9.69E-08	0.30	0.68	*FBXL19-AS1*	Concordant

Supplementary Table 6. Top human orthologs of mouse genes with known mouse knock-out phenotypes of "abnormal IgA levels" (mouse ontology database) and their regional association with IgA levels based on GWAS meta-analysis. The columns include chromosome number, start and end locations of the gene region, strand information for each gene, gene name, and the smallest two-sided P-value (fixed effects GWAS meta-analysis) within each gene region. The results meeting Bonferroni-corrected threshold ($P < 0.05 / 22169 = 2.26E-6$) are listed, 11 of 13 prioritized genes reside within the genome-wide significant loci. Gene names indicated in italics.

CHR	START	STOP	SIZE	STRAND	GENE	P
1	25226001	25291501	65500	-	*RUNX3*	3.42E-92
5	95220801	95297775	76974	-	*ELL2*	7.81E-30
6	31539875	31542100	2225	+	*LTA*	7.15E-26
6	31548335	31550202	1867	-	*LTB*	8.60E-24
6	31543343	31546112	2769	+	*TNF*	8.60E-24
11	65421066	65430443	9377	-	*RELA*	2.57E-22
5	55230924	55290821	59897	-	*IL6ST*	1.41E-20
11	111222980	111250157	27177	-	*POU2AF1*	7.01E-16
14	103243815	103377837	134022	+	*TRAF3*	5.42E-14
16	31212806	31214097	1291	-	*PYCARD*	2.02E-11
6	33540322	33548070	7748	-	*BAK1*	2.92E-11
17	7461608	7464925	3317	+	*TNFSF13*	9.41E-08
2	61108629	61155291	46662	+	*REL*	1.52E-06

Supplementary Table 7. Top human orthologs of mouse genes with known mouse knock-out phenotypes of "abnormal immune tolerance" (mouse ontology database) and their regional association with IgA levels based on GWAS meta-analysis. The columns include chromosome number, start and end locations of the gene region, strand information for each gene, gene name, and the smallest two-sided P-value (fixed effects GWAS meta-analysis) within each gene region. The results meeting Bonferroni-corrected threshold ($P < 0.05 / 22169 = 2.26E\text{-}6$) are listed. Gene names indicated in italics.

CHR	START	STOP	SIZE	STRAND	GENE	P
11	61560108	61564714	4606	+	*FEN1*	2.42E-41
11	61281187	61348344	67157	-	*SYT7*	2.42E-41
6	32158542	32163300	4758	-	*GPSM3*	9.70E-27
6	32627240	32634466	7226	-	*HLA-DQB1*	9.70E-27
6	32812985	32821748	8763	-	*TAP1*	1.90E-26
6	32916390	32920899	4509	-	*HLA-DMA*	6.78E-26
6	31539875	31542100	2225	+	*LTA*	7.15E-26
6	29910246	29913661	3415	+	*HLA-A*	9.93E-26
6	29624757	29640149	15392	+	*MOG*	9.93E-26
6	30457182	30461982	4800	+	*HLA-E*	1.54E-24
6	31582993	31584798	1805	+	*AIF1*	8.60E-24
6	31543343	31546112	2769	+	*TNF*	8.60E-24
6	31949833	31970457	20624	+	*C4A*	4.97E-23
6	31949833	31970458	20625	+	*C4B*	4.97E-23
6	31913720	31919861	6141	+	*CFB*	7.97E-22
5	55230924	55290821	59897	-	*IL6ST*	1.41E-20
9	117546914	117568408	21494	-	*TNFSF15*	1.13E-18
1	173152869	173176471	23602	-	*TNFSF4*	1.06E-16
11	111222980	111250157	27177	-	*POU2AF1*	7.01E-16
12	48103517	48119355	15838	-	*ENDOU*	9.97E-15
12	48436680	48500091	63411	-	*SENP1*	9.97E-15
12	48235319	48298814	63495	-	*VDR*	9.97E-15
14	103243815	103377837	134022	+	*TRAF3*	5.42E-14
16	11348273	11350039	1766	-	*SOCS1*	1.91E-12
6	33540322	33548070	7748	-	*BAK1*	2.92E-11
2	102686835	102796334	109499	+	*IL1R1*	6.19E-10
17	7387697	7417935	30238	+	*POLR2A*	9.41E-08
17	7461608	7464925	3317	+	*TNFSF13*	9.41E-08
2	61108629	61155291	46662	+	*REL*	1.52E-06

Supplementary Table 8. Top human orthologs of mouse genes with known mouse knock-out phenotype of “abnormal response to infection” (mouse ontology database) and their regional association with IgA levels based on GWAS meta-analysis. The columns include chromosome number, start and end locations of the gene region, strand information for each gene, gene name, and the smallest two-sided P-value (fixed effects GWAS meta-analysis) within each gene region. The results meeting Bonferroni-corrected threshold ($P < 0.05 / 22169 = 2.26E-6$) are listed. Gene names indicated in italics.

CHR	START	STOP	SIZE	STRAND	GENE	P
1	25071759	25170815	99056	+	*CLIC4*	3.42E-92
6	30710975	30712327	1352	-	*IER3*	1.86E-31
6	32780539	32784825	4286	-	*HLA-DOB*	9.70E-27
6	32627240	32634466	7226	-	*HLA-DQB1*	9.70E-27
6	32821937	32827628	5691	+	*PSMB9*	1.90E-26
6	32812985	32821748	8763	-	*TAP1*	1.90E-26
6	32916390	32920899	4509	-	*HLA-DMA*	6.78E-26
6	31539875	31542100	2225	+	*LTA*	7.15E-26
6	29910246	29913661	3415	+	*HLA-A*	9.93E-26
6	29794755	29798899	4144	+	*HLA-G*	9.93E-26
6	29523388	29527702	4314	-	*UBD*	9.93E-26
6	30457182	30461982	4800	+	*HLA-E*	1.54E-24
6	31548335	31550202	1867	-	*LTB*	8.60E-24
6	31543343	31546112	2769	+	*TNF*	8.60E-24
6	31949833	31970457	20624	+	*C4A*	4.97E-23
6	31949833	31970458	20625	+	*C4B*	4.97E-23
11	65365225	65381720	16495	-	*MAP3K11*	2.57E-22
11	65421066	65430443	9377	-	*RELA*	2.57E-22
6	31913720	31919861	6141	+	*CFB*	7.97E-22
6	31847535	31865484	17949	-	*EHMT2*	2.08E-21
5	55147206	55218682	71476	+	*IL31RA*	1.41E-20
5	55230924	55290821	59897	-	*IL6ST*	1.41E-20
22	30636435	30642840	6405	-	*LIF*	1.58E-20
11	111222980	111250157	27177	-	*POU2AF1*	7.01E-16
1	27189632	27190947	1315	+	*SFN*	9.94E-15
12	48235319	48298814	63495	-	*VDR*	9.97E-15
16	11348273	11350039	1766	-	*SOCS1*	1.91E-12
16	31271287	31344213	72926	+	*ITGAM*	2.02E-11
16	31212806	31214097	1291	-	*PYCARD*	2.02E-11
2	102686835	102796334	109499	+	*IL1R1*	6.19E-10
2	102927961	102968497	40536	+	*IL1RL1*	6.19E-10
9	117096432	117156685	60253	-	*AKNA*	2.22E-09
17	7076750	7082883	6133	-	*ASGR1*	9.41E-08
17	7743234	7758118	14884	+	*KDM6B*	9.41E-08
17	7452374	7461207	8833	+	*TNFSF12*	9.41E-08
13	50571142	50592603	21461	+	*TRIM13*	2.86E-07
19	55417507	55424439	6932	+	*NCR1*	5.01E-07
7	128577990	128590096	12106	+	*IRF5*	1.06E-06
1	27938800	27961727	22927	-	*FGR*	1.23E-06
2	143886898	144525921	639023	+	*ARHGAP15*	1.50E-06
2	61108629	61155291	46662	+	*REL*	1.52E-06

Supplementary Table 9. The associations of top SNPs from GWAS for IgA levels in GWAS for IgA nephropathy. Among 31 independent alleles associated with increased IgA levels, 12 had nominal association with increased risk of IgA nephropathy at two-sided *P*<0.05 (all with concordant effects). The P-values correspond to fixed effects meta-analyses for IgA levels and IgA nephropathy, respectively. Gene names indicated in italics.

Locus	CHR	BP	SNP	IgA level GWAS			IgA Nephropathy GWAS			Direction
				Risk Allele	BETA	P-value	Risk Allele	BETA	P-value	
RUNX3	1	25291697	rs188468174	C	0.876	3.42E-92	C	0.009	9.68E-01	-
TNFSF4	1	173163568	rs7518129	G	0.056	1.06E-16	G	0.118	6.71E-07	Concordant
IL1R1	2	102689031	rs13427957	C	0.040	6.19E-10	C	0.024	2.48E-01	-
ELL2	5	95277555	rs3777175	G	0.084	7.81E-30	A	0.005	8.73E-01	-
ANKRD55	5	55438580	rs6859219	C	0.073	1.41E-20	C	0.167	1.96E-07	Concordant
HLA	6	31106893	rs1265094	A	0.076	1.86E-31	A	0.047	2.70E-02	Concordant
RUNX2	6	45526470	rs1200427	T	0.059	6.85E-14	T	0.045	2.32E-01	-
FILNC1	6	139975943	rs17069163	T	0.050	5.94E-11	C	0.007	7.68E-01	-
ZP3,SSC4D	7	76034150	rs55722505	C	0.048	8.61E-14	C	0.034	1.80E-01	-
TNFSF8	9	117692882	rs3181356	T	0.069	1.13E-18	T	0.163	1.03E-04	Concordant
FADS2	11	61595564	rs968567	T	0.116	2.42E-41	T	0.050	1.21E-01	-
OVOL1	11	65555524	rs10896045	A	0.066	2.57E-22	A	0.168	4.77E-13	Concordant
POU2AF1	11	111267394	rs4938518	T	0.056	7.01E-16	T	0.016	4.66E-01	-
HDAC7	12	48214825	rs7487637	G	0.054	9.97E-15	G	0.100	6.78E-03	Concordant
SH2B3	12	111833788	rs10774624	G	0.050	1.37E-13	G	0.092	1.23E-02	Concordant
RCOR1,TRAF3	14	103239630	rs12147883	C	0.048	5.42E-14	C	0.042	6.40E-02	-
LITAF	16	11717832	rs113962704	T	0.054	1.91E-12	T	0.055	1.96E-01	-
CTF1	16	30916129	rs1458201	A	0.052	2.02E-11	A	0.019	4.61E-01	-
HORMAD2	22	30448399	rs193473	A	0.067	1.58E-20	A	0.139	9.07E-07	Concordant

Supplementary Table 10. Colocalization analysis of GWAS loci for IgA levels with GWAS signals for IgA nephropathy PP1: probability of the locus being associated with only IgA levels; PP2: probability of the locus being associated with only IgA nephropathy; PP3: probability of not sharing the same casual variant at the locus; and PP4: probability of sharing the same causal variant at the locus between the two traits. High probability of sharing causal variants was evident for five loci (*). Gene names indicated in italics.

Locus	#SNPs	CHR	Start	End	PP1	PP2	PP3	PP4
OVOL1,RELA	2013	11	65156514	65955511	0.00	0.00	0.00	1.00*
ANKRD55,IL6ST	2804	5	55039671	55838485	0.00	0.00	0.00	1.00*
HORMAD2,LIF	2224	22	30048571	30848388	0.00	0.00	0.02	0.98*
TNFSF4,TNFSF18	2021	1	172763639	173563418	0.00	0.00	0.04	0.96*
SH2B3	1031	12	111438001	112233495	0.09	0.00	0.02	0.90*
HLA	15091	6	30706942	31506854	0.00	0.00	1.00	0.00
CTF1	819	16	30516148	31314599	0.01	0.00	0.99	0.00
RCOR1,TRAF3	2415	14	102840160	103639456	0.03	0.00	0.89	0.08
TNFSF8	3254	9	117293051	118092515	0.01	0.00	0.83	0.16
RUNX2	2826	6	45127152	45926460	0.38	0.00	0.61	0.01
LITAF	3543	16	11317993	12117447	0.85	0.00	0.14	0.02
POU2AF1	2328	11	110867986	111666128	0.86	0.00	0.13	0.01
IL1R1	2715	2	102289430	103088777	0.87	0.00	0.12	0.02
RUNX3	1953	1	24891715	25689406	0.85	0.00	0.11	0.03
FILNC1	2459	6	139576015	140375356	0.90	0.00	0.09	0.01
FADS2,FADS1	2288	11	61196289	61995513	0.89	0.00	0.08	0.03
HDAC7, VDR	3153	12	47814851	48614567	0.72	0.00	0.06	0.22
ELL2	1753	5	94878263	95677384	0.94	0.00	0.05	0.01
ZP3,SSC4D	2200	7	75634687	76433936	0.94	0.00	0.05	0.02

Supplementary Table 11. Colocalization analysis of GWAS loci for IgA levels with GWAS signals for tonsillectomy
PP1: probability of the locus being associated with only IgA levels; PP2: probability of the locus being associated with only tonsillectomy; PP3: probability of not sharing the same casual variant at the locus; and PP4: probability of sharing the same causal variant at the locus between the two traits. High probability of sharing causal variants was evident for two loci (*). Gene names indicated in italics.

Locus	#SNPs	CHR	Start	End	PP1	PP2	PP3	PP4
SH2B3	1542	12	111438001	112233495	0.00	0.00	0.01	0.99*
HORMAD2,LIF	2494	22	30048571	30848388	0.00	0.00	0.22	0.78*
HLA	13524	6	30706942	31506854	0.00	0.00	1.00	0.00
CTF1	1119	16	30516148	31314599	0.09	0.00	0.91	0.00
RCOR1,TRAF3	2307	14	102840084	103639456	0.52	0.00	0.47	0.01
LITAF	3549	16	11317993	12117566	0.58	0.00	0.42	0.00
IL1R1	2920	2	102289430	103088777	0.50	0.00	0.39	0.11
FILNC1	2607	6	139576015	140374954	0.83	0.00	0.16	0.00
TNFSF8	3268	9	117293051	118092515	0.90	0.00	0.08	0.02
TNFSF4,TNFSF18	2430	1	172763639	173563418	0.93	0.00	0.06	0.01
FADS2,FADS1	2581	11	61196289	61995513	0.94	0.00	0.05	0.00
POU2AF1	2516	11	110867986	111666128	0.93	0.00	0.05	0.02
RUNX3	2213	1	24891715	25688276	0.93	0.00	0.04	0.02
HDAC7, VDR	3198	12	47814851	48614567	0.96	0.00	0.04	0.00
OVOL1,RELA	2273	11	65156283	65955511	0.96	0.00	0.04	0.00
ANKRD55,IL6ST	2964	5	55039671	55838571	0.96	0.00	0.03	0.00
RUNX2	2971	6	45127152	45926460	0.97	0.00	0.03	0.00
ELL2	2170	5	94878263	95677384	0.97	0.00	0.02	0.00
ZP3,SSC4D	2082	7	75634687	76433287	0.98	0.00	0.02	0.00

Supplementary Table 12. Genome-wide genetic correlations between serum IgA level and related traits or disorders without HLA region (left) and with HLA region (right). All analyses are performed with LDSC software, h2: SNP-based heritability, rg: genetic correlation coefficient, se: standard error, z: z-score, p: p-value for a two-sided test of genetic correlation by LD score regression.

Trait	Reference	h2	Correlations with HLA region				Correlations without HLA region			
			rg	se	z	p	rg	se	z	p
IgA deficiency	Bronson et al. Nat Genet 2016	1.000	-0.38	0.10	3.67	2.00E-04	0.20	0.10	2.12	3.44E-02
Celiac Disease	Zhou et al. Nat Genet 2018	0.054	-0.27	0.05	-5.60	2.16E-08	-0.21	0.08	-2.71	6.80E-03
Primary Sclerosing Cholangitis	Ji et al. Nat Genet 2017	0.682	-0.27	0.15	-1.77	7.74E-02	-0.16	0.08	-2.01	4.46E-02
Membranous Nephropathy	Xie et al. Nat Comm 2020	0.600	-0.20	0.07	-2.96	3.10E-03	-0.14	0.15	-0.99	3.24E-01
Crohn's Disease	De Lange et al. Nat Genet 2017	0.261	-0.15	0.06	-2.55	1.09E-02	-0.19	0.07	-2.79	5.30E-03
Type 1 Diabetes	Zhou et al. Nat Genet 2018	0.011	-0.13	0.23	-0.57	5.69E-01	-0.07	0.16	-0.46	6.42E-01
Systemic Lupus Erythematosus	Bentham et al. Nat Genet 2015	0.491	-0.12	0.13	-0.88	3.76E-01	-0.04	0.09	-0.40	6.89E-01
Inflammatory Bowel Diseases	De Lange et al. Nat Genet 2017	0.183	-0.12	0.05	-2.23	2.58E-02	-0.13	0.06	-2.08	3.76E-02
Ulcerative Colitis	De Lange et al. Nat Genet 2017	0.165	-0.06	0.07	-0.90	3.70E-01	-0.03	0.08	-0.41	6.80E-01
Allergy	Ferreira et al. Nat Genet 2017	0.031	0.08	0.04	1.95	5.06E-02	0.07	0.06	1.14	2.55E-01
Rheumatoid Arthritis	Okada et al. Nature 2014	0.344	0.11	0.08	1.33	1.83E-01	0.15	0.06	2.41	1.58E-02
Multiple Sclerosis	Int. MS Genetics Consortium. Science 2019	0.250	0.24	0.09	2.75	5.90E-03	0.05	0.17	0.28	7.83E-01
IgA Nephropathy	Kiryluk et al. accomplishing paper	0.047	0.31	0.10	3.08	2.10E-03	0.35	0.11	3.07	2.10E-03
bacterial meningitis	Tian et al. Nat Comm 2017	0.003	-0.35	0.13	-2.65	8.10E-03	-0.47	0.16	-2.82	4.80E-03
Positive TB Test	Tian et al. Nat Comm 2017	0.009	-0.32	0.28	-1.15	2.52E-01	-0.17	0.30	-0.58	5.61E-01
Shingles	Tian et al. Nat Comm 2017	0.013	-0.23	0.11	-1.99	4.71E-02	-0.46	0.17	-2.63	8.60E-03
Measles	Tian et al. Nat Comm 2017	0.002	-0.22	0.65	-0.34	7.34E-01	-0.20	0.22	-0.92	3.57E-01
Rubella	Tian et al. Nat Comm 2017	0.005	-0.22	0.10	-2.28	2.29E-02	-0.27	0.17	-1.59	1.12E-01
Scarlet fever	Tian et al. Nat Comm 2017	0.007	-0.19	0.13	-1.47	1.43E-01	-0.11	0.13	-0.84	4.00E-01
Mononucleosis	Tian et al. Nat Comm 2017	0.004	-0.16	0.29	-0.57	5.71E-01	-0.54	0.31	-1.73	8.41E-02
Mumps	Tian et al. Nat Comm 2017	0.005	-0.11	0.16	-0.72	4.72E-01	0.01	0.17	0.07	9.42E-01
Cold sores	Tian et al. Nat Comm 2017	0.005	-0.08	0.26	-0.31	7.57E-01	0.15	0.22	0.70	4.84E-01
Myringotomy	Tian et al. Nat Comm 2017	0.006	-0.07	0.09	-0.71	4.75E-01	-0.01	0.13	-0.08	9.38E-01
Chicken pox	Tian et al. Nat Comm 2017	0.008	-0.06	0.08	-0.76	4.46E-01	0.01	0.12	0.09	9.25E-01
Hepatitis B	Tian et al. Nat Comm 2017	0.006	-0.02	0.13	-0.14	8.86E-01	0.20	0.12	1.70	8.95E-02
Childhood Ear Infections	Tian et al. Nat Comm 2017	0.018	-0.01	0.07	-0.15	8.79E-01	0.06	0.09	0.63	5.26E-01
Yeast infections	Tian et al. Nat Comm 2017	0.009	0.00	0.07	0.07	9.45E-01	0.01	0.09	0.10	9.20E-01
UTI Frequency	Tian et al. Nat Comm 2017	0.011	0.01	0.08	0.10	9.19E-01	-0.02	0.10	-0.15	8.78E-01
Colds Last Year	Tian et al. Nat Comm 2017	0.008	0.02	0.08	0.23	8.17E-01	-0.03	0.11	-0.26	7.95E-01
Rheumatic Fever	Tian et al. Nat Comm 2017	0.004	0.03	0.14	0.20	8.39E-01	0.19	0.15	1.27	2.04E-01
Strep Throat	Tian et al. Nat Comm 2017	0.007	0.04	0.20	0.18	8.60E-01	0.24	0.15	1.61	1.07E-01
Tonsillectomy	Tian et al. Nat Comm 2017	0.028	0.08	0.11	0.75	4.56E-01	0.20	0.08	2.48	1.31E-02
Plantar Warts	Tian et al. Nat Comm 2017	0.014	0.09	0.27	0.33	7.39E-01	-0.24	0.12	-1.96	4.95E-02
Hepatitis A	Tian et al. Nat Comm 2017	0.003	0.12	0.21	0.54	5.86E-01	-0.06	0.17	-0.36	7.19E-01
Chronic Sinus Infections	Tian et al. Nat Comm 2017	0.004	0.12	0.11	1.12	2.62E-01	0.05	0.15	0.35	7.28E-01
Pneumonia	Tian et al. Nat Comm 2017	0.014	0.18	0.11	1.63	1.03E-01	0.08	0.11	0.77	4.44E-01
Serum LDL	Willer et al. Nat Gen 2013	0.035	-0.10	0.07	-1.44	1.49E-01	-0.14	0.09	-1.51	1.32E-01
Serum Cholesterol	Willer et al. Nat Gen 2013	0.047	-0.09	0.05	-1.73	8.41E-02	-0.17	0.09	-1.83	6.78E-02
Height	Wood et al. Nat Genet 2014	0.413	-0.05	0.04	-1.09	2.77E-01	-0.03	0.05	-0.54	5.87E-01
Serum Triglycerides	Willer et al. Nat Gen 2013	0.054	-0.02	0.10	-0.16	8.71E-01	0.03	0.11	0.22	8.27E-01
eGFR Creatinine	Wuttke et al. Nat Genet 2019	0.062	0.02	0.07	0.35	7.29E-01	0.01	0.09	0.14	8.89E-01
eGFR Cystatine C	Wuttke et al. Nat Genet 2019	0.086	0.02	0.09	0.27	7.90E-01	0.06	0.13	0.48	6.34E-01
Coronary Artery Disease	Nikpay et al. Nat Genet 2015	0.040	0.05	0.04	1.27	2.02E-01	0.08	0.05	1.73	8.36E-02
Essential Hypertension	Zhou et al. Nat Genet 2018	0.033	0.05	0.05	1.12	2.65E-01	0.09	0.05	1.77	7.74E-02
Albuminuria	Haas et al. Am J Hum Genet 2018	0.024	0.07	0.05	1.63	1.03E-01	0.03	0.06	0.54	5.90E-01
Chronic Kidney Disease	Wuttke et al. Nat Genet 2019	0.012	0.11	0.16	0.69	4.90E-01	0.20	0.32	0.62	5.35E-01
Body Mass Index	Locke et al. Nature 2015	0.163	0.13	0.06	2.38	1.71E-02	0.13	0.06	2.15	3.16E-02
Type 2 Diabetes	Xue et al. Nat Comm 1018	0.026	0.17	0.07	2.55	1.07E-02	0.18	0.07	2.52	1.16E-02

Supplementary Table 13. Meta-phenome-wide association study (Meta-PheWAS) for the genome-wide polygenic score (GPS) for serum IgA levels across eMERGE-III and UKBB datasets. Only the top associations exceeding the phenome-wide significance threshold are listed for the GPS **a)** with and **b)** without the HLA region. OR: odds ratio per standard deviation of the GPS; SE: standard error; all associations are adjusted for age, sex, site/cohort, genotyping batch, and ancestry, eEMRGE-III participants included in the GWAS for IgA levels were excluded from the analysis. Two-sided P-value corresponds to the fixed effects meta-analysis across the two biobanks based on logistic regression adjusted for age, sex, site, genotyping batch, and principal components of ancestry

a) GPS with HLA

Phenotype	Description	Phenotype Group	Beta	OR	SE	P value	N cases	N controls
557.1	Celiac disease	digestive	-0.621	0.537	0.019	4.62E-227	2623	385965
244.4	Hypothyroidism NOS	endocrine/metabolic	-0.060	0.941	0.007	1.77E-19	29802	460255
275.1	Disorders of iron metabolism	hematopoietic	0.273	1.313	0.030	3.97E-19	1333	493272
244	Hypothyroidism	endocrine/metabolic	-0.058	0.944	0.007	1.03E-18	31543	460255
242	Thyrotoxicosis with or without goiter	endocrine/metabolic	-0.125	0.882	0.017	1.55E-13	4473	460255
250.1	Type 1 diabetes	endocrine/metabolic	-0.093	0.911	0.014	8.22E-12	7497	447124
696	Psoriasis and related disorders	dermatologic	-0.097	0.908	0.015	1.69E-10	5369	465843
242.1	Graves' disease	endocrine/metabolic	-0.202	0.817	0.032	1.74E-10	1316	460255
696.4	Psoriasis	dermatologic	-0.098	0.907	0.015	2.32E-10	4996	465843
593	Hematuria	genitourinary	0.040	1.041	0.007	1.04E-08	26620	440047
250.13	Type 1 diabetes with ophthalmic manifestations	endocrine/metabolic	-0.184	0.832	0.033	3.18E-08	1106	447124
695.3	Rosacea	dermatologic	0.132	1.141	0.024	4.45E-08	2666	474313
555	Inflammatory bowel disease and other gastroenteritis and colitis	digestive	0.068	1.071	0.013	5.83E-08	7671	385965
696.41	Psoriasis vulgaris	dermatologic	-0.093	0.912	0.017	1.01E-07	4014	465843
335	Multiple sclerosis	neurological	0.119	1.126	0.024	4.37E-07	2134	460061
454.1	Varicose veins of lower extremity	circulatory system	0.041	1.042	0.008	6.63E-07	17180	422020
555.2	Ulcerative colitis	digestive	0.073	1.076	0.015	1.41E-06	5187	385965
454	Varicose veins	circulatory system	0.039	1.040	0.008	1.54E-06	18258	422020
250.11	Type 1 diabetes with ketoacidosis	endocrine/metabolic	-0.230	0.794	0.048	1.75E-06	523	447124
695.4	Lupus (localized and systemic)	dermatologic	-0.143	0.867	0.030	2.51E-06	1592	471987
555.21	Ulcerative colitis (chronic)	digestive	0.141	1.151	0.031	4.78E-06	1415	385965
251.1	Hypoglycemia	endocrine/metabolic	-0.101	0.904	0.023	7.12E-06	2511	434768
695.42	Systemic lupus erythematosus	dermatologic	-0.146	0.864	0.033	7.27E-06	1402	471987
250	Diabetes mellitus	endocrine/metabolic	-0.025	0.975	0.006	7.68E-06	47177	447124
706	Diseases of sebaceous glands	dermatologic	0.037	1.037	0.008	8.68E-06	21288	474164
580.12	Non-proliferative glomerulonephritis	genitourinary	-0.238	0.788	0.054	9.63E-06	556	457242
695.21	Dermatitis herpetiformis	dermatologic	-0.550	0.577	0.124	9.97E-06	69	474313
285	Other anemias	hematopoietic	-0.028	0.972	0.007	1.47E-05	37100	444319
371	Inflammation of the eye	sense organs	0.055	1.057	0.013	1.92E-05	9889	473293
250.12	Type 1 diabetes with renal manifestations	endocrine/metabolic	-0.212	0.809	0.050	2.17E-05	599	447124
740	Osteoarthrosis	musculoskeletal	-0.018	0.982	0.004	3.00E-05	81698	418302

b) GPS without HLA

Phenotype	Description	Phenotype Group	Beta	OR	SE	P value	N cases	N controls
557.1	Celiac disease	digestive	-0.153	0.858	0.022	5.90E-12	388588	2623
278.11	Morbid obesity	endocrine/metabolic	0.083	1.087	0.020	3.00E-05	467399	8411
