# Supplementary material for: Genetic regulation of serum IgA levels and susceptibility to common immune, infectious, kidney, and cardio-metabolic traits
Source: Nat Commun. 2022 Nov 11;13:6859. doi: 10.1038/s41467-022-34456-6 (PMC9651905; doi:10.1038/s41467-022-34456-6)
Supplement: Supplementary file 3 — Description of Additional Supplementary Files [file 41467_2022_34456_MOESM3_ESM.pdf]

Description of Additional Supplementary Files

File Name: Supplementary Data 1
Description: **Genetic association results for the 20 genome-wide significant associations by cohort for all 17 cohorts meta-analyzed in this study.** P-values: two-sided Wald test based on linear regression of individual variants against serum IgA levels controlling for age, sex, and principal components of ancestry. Gene names indicated in italics.

File Name: Supplementary Data 2
Description: We interrogated genome-wide significant GWAS loci for IgA levels against all previously studied GWAS phenotypes based on the GWAS catalogue; any reported trait associations for SNPs in LD ($R^2 > 0.5$) with the top variant at each GWAS locus are listed; the *SH2B3* locus exhibited the greatest range of pleiotropic associations. P-values correspond to a two-sided test for genome-wide genetic correlation by LDSC. Gene names indicated in italics.
