# Supplementary material for: Genetic regulation of serum IgA levels and susceptibility to common immune, infectious, kidney, and cardio-metabolic traits
Source: Nat Commun. 2022 Nov 11;13:6859. doi: 10.1038/s41467-022-34456-6 (PMC9651905; doi:10.1038/s41467-022-34456-6)
Supplement: Supplementary file 6 — Reporting Summary [file 41467_2022_34456_MOESM6_ESM.pdf]

nature portfolio

Corresponding author(s): Krzysztof Kiryluk

Last updated by author(s): Oct 14, 2022

Reporting Summary

Nature Portfolio wishes to improve the reproducibility of the work that we publish. This form provides structure for consistency and transparency in reporting. For further information on Nature Portfolio policies, see our Editorial Policies and the Editorial Policy Checklist.

Statistics

For all statistical analyses, confirm that the following items are present in the figure legend, table legend, main text, or Methods section.

n/a	Confirmed	
☐	☒	The exact sample size (*n*) for each experimental group/condition, given as a discrete number and unit of measurement
☐	☒	A statement on whether measurements were taken from distinct samples or whether the same sample was measured repeatedly
☐	☒	The statistical test(s) used AND whether they are one- or two-sided<br>*Only common tests should be described solely by name; describe more complex techniques in the Methods section.*
☐	☒	A description of all covariates tested
☐	☒	A description of any assumptions or corrections, such as tests of normality and adjustment for multiple comparisons
☐	☒	A full description of the statistical parameters including central tendency (e.g. means) or other basic estimates (e.g. regression coefficient) AND variation (e.g. standard deviation) or associated estimates of uncertainty (e.g. confidence intervals)
☐	☒	For null hypothesis testing, the test statistic (e.g. *F*, *t*, *r*) with confidence intervals, effect sizes, degrees of freedom and *P* value noted<br>*Give P values as exact values whenever suitable.*
☐	☒	For Bayesian analysis, information on the choice of priors and Markov chain Monte Carlo settings
☒	☐	For hierarchical and complex designs, identification of the appropriate level for tests and full reporting of outcomes
☐	☒	Estimates of effect sizes (e.g. Cohen's *d*, Pearson's *r*), indicating how they were calculated

Our web collection on statistics for biologists contains articles on many of the points above.

Software and code

Policy information about availability of computer code

Data collection	No custom software or algorithms were used.
Data analysis	No custom software or algorithms were used. Only open source software packages were used for data analysis, including the following: R version 3.4 (CRAN), PLINK v1.9, METAL version 2011-03-25, TransMeta, EAGLE v2.3, MINIMAC3, KING, GCTA version 1.92.0beta, Cytoscape v3.6, ANNOVAR, FlashPCA, COLOC, LDPred, LDSC, DEPICTv1 release 194, TwoSampleMR , PheWAS (R), ImPG version 1.0,

For manuscripts utilizing custom algorithms or software that are central to the research but not yet described in published literature, software must be made available to editors and reviewers. We strongly encourage code deposition in a community repository (e.g. GitHub). See the Nature Portfolio guidelines for submitting code & software for further information.

Data

Policy information about availability of data

All manuscripts must include a data availability statement. This statement should provide the following information, where applicable:

- Accession codes, unique identifiers, or web links for publicly available datasets
- A description of any restrictions on data availability
- For clinical datasets or third party data, please ensure that the statement adheres to our policy

The MESA SHARe genotype and phenotype data (including serum IgA levels measured in this study) are available through dbGAP, accession number phs000209.v13.p3 (https://www.ncbi.nlm.nih.gov/projects/gap/cgi-bin/study.cgi?study_id=phs000209.v13.p3). The Electronic Medical Records and Genomics-III

(eMERGE-III) imputed genotype and phenotype data are available through dbGAP, accession number: phs001584.v2.p2 (https://www.ncbi.nlm.nih.gov/projects/gap/cgi-bin/study.cgi?study_id=phs001584.v2.p2). The UK Biobank genotype and phenotype data are available through the UK Biobank web portal. Genotype data for other cohorts are available through dbGAP, accession number: phs000431.v3.p1 (link in progress). The 1000 Genomes data are available publicly through https://www.internationalgenome.org/category/data-access/. GWAS summary statistics are available for download from http://www.columbiamedicine.org/divisions/kiryluk/study_gwas_stat.php.

Human research participants

Policy information about studies involving human research participants and Sex and Gender in Research.

Reporting on sex and gender	We included both male and famale participants in all analyses, and adjusted our GWAS statistics using the sex covariate imputed from genetic data.
Population characteristics	This is a population based study based on the analysis of 17 population-based cohorts. Individual cohorts were recruited across multiple centers as described in the Methods. All participants were consented for genetic studies. We used genetic principal component-defined ancestry to further sub-stratify our cohorts into ancestry specific sub-cohorts in order to adequately control for population stratification in GWAS analyses, and to enable detection of ancestry-specific effects.
Recruitment	The recruitment was performed by individual studies as previously described and referenced in the Methods section. We included adult individuals of all racial/ethnic backgrounds and of both sexes. All participants provided informed consent to participate in genetic studies.
Ethics oversight	The Institutional Review Board of Columbia University approved our studies under the following protocol numbers: IRB-AAAC7385 (primary analysis), IRB-AAAQ9205 (eMERGE-III analysis), IRB-AAAC9458 (MESA SHARe analysis), and IRB-AAAS3500 (UK Biobank analysis).

Note that full information on the approval of the study protocol must also be provided in the manuscript.

Field-specific reporting

Please select the one below that is the best fit for your research. If you are not sure, read the appropriate sections before making your selection.

☒ Life sciences ☐ Behavioural & social sciences ☐ Ecological, evolutionary & environmental sciences

For a reference copy of the document with all sections, see nature.com/documents/nr-reporting-summary-flat.pdf

Life sciences study design

All studies must disclose on these points even when the disclosure is negative.

Sample size	To maximize our power for discovery, we aimed to include all available cohorts with genotypes and relevant phenotypes (serum IgA levels), including all published datasets. To identify all published datasets, we searched the literature to identify all prior GWAS studies for serum IgA levels and requested GWAS summary statistics for joint meta-analyses. In the end, we conducted a global multiethnic meta-analysis of 16 cohorts with genome-wide data for 22,229 diverse study participants with 4,699 suggestive association signals (P<10-6) from the previous GWAS by DeCode Genetics (19,034 participants of North European ancestry). There are no additional datasets that were available to us to increase the sample size further, thus the availability of samples and data determined the final sample size rather than the formal power calculation.
Data exclusions	Only suggestive association signals (P<10-6) were available for meta-analysis from the previous GWAS by DeCode Genetics (DeCODE declined to share genome-wide summary statistics for their previously published study).
Replication	The association signals were examined for replication in each individual independent cohort (17 independent cohorts in total) as well as by each continental ancestral group. For each of the genome-wide significant loci, we additionally assessed heterogeneity of associations across all cohorts and ancestries, and robustness of signals to alternative meta-analysis models. The genotyping (SNP typing) and phenotyping (serum IgA levels measurements) was performed only once for each cohort.
Randomization	Not applicable, randomization is not relevant to our study design since this is a population-based observational study.
Blinding	Not applicable, blinding is not relevant to our study design since this is a population-based observational study.

Reporting for specific materials, systems and methods

We require information from authors about some types of materials, experimental systems and methods used in many studies. Here, indicate whether each material, system or method listed is relevant to your study. If you are not sure if a list item applies to your research, read the appropriate section before selecting a response.

Materials & experimental systems

n/a	Involved in the study
☐	☒ Antibodies
☒	☐ Eukaryotic cell lines
☒	☐ Palaeontology and archaeology
☒	☐ Animals and other organisms
☒	☐ Clinical data
☒	☐ Dual use research of concern

Methods

n/a	Involved in the study
☒	☐ ChIP-seq
☒	☐ Flow cytometry
☒	☐ MRI-based neuroimaging

Antibodies

Antibodies used	Mouse monoclonal Ab to Human IgA horseradish peroxidase conjugate
Validation	Mouse monoclonal Ab to Human IgA horseradish peroxidase conjugate (Abcam Bio ab7383) was extensively tested by the manufacturer for use in sandwich ELISA (https://www.abcam.com/hrp-human-iga-antibody-1h9-ab7383.html) and was validated by us against serially diluted human IgA standard.

March 2021
